# COVID-19 vaccination immune response in patients with solid organ and haematologic malignancies: call for active monitoring

**DOI:** 10.3332/ecancer.2021.1284

**Published:** 2021-09-07

**Authors:** Laudy Chehade, Jad Zeitoun, Rachelle Bejjany, Maya Charafeddine, Firas Kreidieh, Mona Hassan, Ali Taher, Nagi El Saghir, Ali Shamseddine, Ziad Salem, Sally Temraz, Arafat Tfayli, Hazem Assi, Ali Bazarbachi, Jean El Cheikh, Iman Abou Dalle, Nesrine Rizk, Rami Mahfouz, Deborah Mukherji

**Affiliations:** 1 Naef K. Basile Cancer Institute, American University of Beirut Medical Center, PO Box 11-0236, Riad El Solh, Beirut 1107 2020, Lebanon; 2 Division of Infectious Diseases, Department of Internal Medicine, American University of Beirut Medical Center, PO Box 11-0236, Riad El Solh, Beirut 1107 2020, Lebanon; 3 Department of Pathology and Laboratory Medicine, American University of Beirut Medical Center, PO Box 11-0236, Riad El Solh, Beirut 1107 2020, Lebanon

**Keywords:** COVID-19, vaccine, chemotherapy, immune response

## Abstract

Vaccines against COVID-19 have demonstrated a remarkable efficacy in decreasing hospitalisations and deaths; however, clinical trials leading to vaccine approvals did not include immunocompromised individuals such as patients receiving antineoplastic therapies. Emerging data suggest that patients on active anti-cancer therapy may have a reduced immune response to COVID-19 vaccination compared to the general population and may be at greater risk of COVID-19 infection as measures to reduce transmission in the community are relaxed. We report preliminary data from the American University of Beirut Medical Center in Lebanon demonstrating relatively low seroconversion rates. Of 36 patients on active anti-cancer therapy who had received two doses of vaccine, 17% were negative for Severe Acute Respiratory Syndrome Coronavirus 2 (SARS-CoV-2) anti-spike IgG. These results highlight the importance of maintaining strict precautionary measures against COVID-19 in patients on immunosuppressive treatment. There is an urgent need for active monitoring of immune response post-vaccination in prospective studies involving populations from diverse resource settings.

Despite the lack of data on the efficacy of COVID-19 vaccination in patients on anti-neoplastic therapy from registration trials, vaccination in this vulnerable population is strongly recommended due to the increased risk of severe COVID-19 infection [1].

Data from case series in high-income countries suggest that the proportion of patients with cancer on active therapy who do not develop antibodies after two doses of the Pfizer BNT162b2 mRNA COVID-19 vaccine ranges from 6% to 10% [2, 3]. To date, we have limited data on the duration of immune response in patients on active anti-cancer therapy or the potential benefit of vaccine booster doses in patients with an insufficient or short-lived immune response. Ehmsen *et al* [4] recently reported a three-fold reduction approximately in antibody titers after a period of 12 weeks post vaccination compared to 5 weeks post vaccination; however, more data are needed to support this finding. As COVID-19 infection control measures in the community are relaxed, there is a concern that immunosuppressed patients remain at increased risk of COVID-19 infection despite being offered vaccination.

The COVID-19 and Cancer Taskforce has previously recommended a global vaccine-response monitoring programme and a draft protocol has been published [5]. We report preliminary data from the American University of Beirut Medical Center in Lebanon, where the economic crisis and currency depreciation have made access to essential medications and other resources to support the healthcare system increasingly limited.

Following institutional review board approval of the protocol, patients diagnosed with a solid organ or haematological malignancy who were on active systemic treatment at the time of planned vaccination (chemotherapy, targeted therapy, immune checkpoint inhibitor therapy, endocrine therapy) at the American University of Beirut Medical Center, Beirut, Lebanon, were included. Following written informed consent, patients were tested for COVID-19 immunoglobulin G (IgG) using a chemiluminescent microparticle immunoassay developed by Abbot Diagnostics, which measures IgG antibodies against the spike receptor-binding domain of SARS-CoV-2 (cutoff for positive test is 50 AU/mL). This assay has a specificity of 99.6% and a sensitivity of 99.35% [6].

Fifty patients were recruited with a median age of 64.5 years, 48% were males and 52% were females. Seven patients (14%) had COVID-19 infection prior to vaccination. 12% (*n* = 6) of the patients had a haematological malignancy and the rest had a solid organ tumour, with the following distribution: gastrointestinal 42%, breast 24%, lung 16% and genitourinary 6% (Table 1). All patients have received at least one dose of the BNT162b2 mRNA vaccine except one patient who received ChAdOx1 (two doses), one patient who received Gam-COVID-Vac (two doses) and one patient who received both BBIBP-CorV and BNT162b2 vaccines. Six patients (12%) had negative IgG titers after receiving two doses of vaccine.

Out of 14 patients who have received only one dose of the vaccine, 7 had negative IgG results (seroconversion rate following one dose is 50%). Six out of these 7 patients had no previous COVID-19 infection and only one a prior infection (Figure 1).

Out of 36 patients who have received two doses, six had negative IgG results (17%) and none of them had a prior COVID-19 infection (seroconversion rate following two doses is 83%) (Figure 2). Out of these patients, two were on chemotherapy, one was on chemotherapy and anti-VEGF, two were on Anti-CD 20 therapy and one was taking bispecific T cell engager. One patient with lymphoma on Rituximab had COVID-19 infection 24 days after the second dose of the ChAdOx1 vaccine. This patient had a negative pre-infection IgG test.

## Conclusions

To the best of author’s knowledge, this is the first report of COVID-19 immune response in cancer patients in a limited resource setting. In our study, seroconversion rates were 50% following the first vaccine dose and 83% after the second dose, slightly lower than data from case series reported from high-income countries [7–10]. A bigger sample size from multiple sources is needed to infer correlations with the type of malignancy, treatment and vaccine type. It is important to note that similar to other studies, patients receiving Anti-CD 20 drugs did not mount an adequate antibody response, likely due to B-cell depleting action. Antibody response is a surrogate marker of vaccine efficacy; however, it does not entirely confer the level of protection provided, part of which is mediated by T-cells [11]. The need to maintain safety precautions post vaccination should be reinforced for all immunosuppressed patients. This is particularly important in low-income countries with lower population vaccination rates compared to high-income countries. Further data from longitudinal studies are required to monitor the response to COVID-19 vaccination in patients with cancer on therapy in different resource settings. Prospectively evaluating the requirement for and efficacy of vaccine booster doses is urgently required.

## Funding

This study was funded internally by the Naef K. Basile Cancer Institute. Dr Mukherji is a member of the Cancer and COVID-19 Taskforce and is funded through UK Research and Innovation as part of the Global Challenges Research Fund; Research for Health in Conflict in the Middle East and North Africa (R4HC-MENA) project, grant number ES/P010962/1.

## Conflicts of interest

The authors do not report any relevant conflicts of interest.

## Figures and Tables

**Figure 1.**
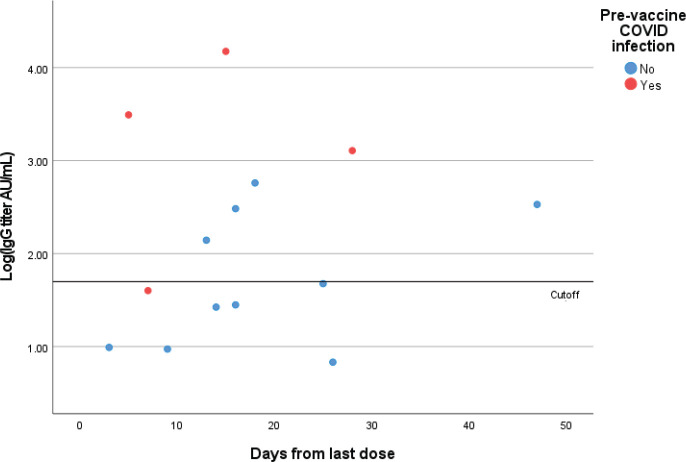
Quantitative IgG titers (logarithmic scale) for patients who received one dose of the vaccine. Each point represents one patient.

**Figure 2.**
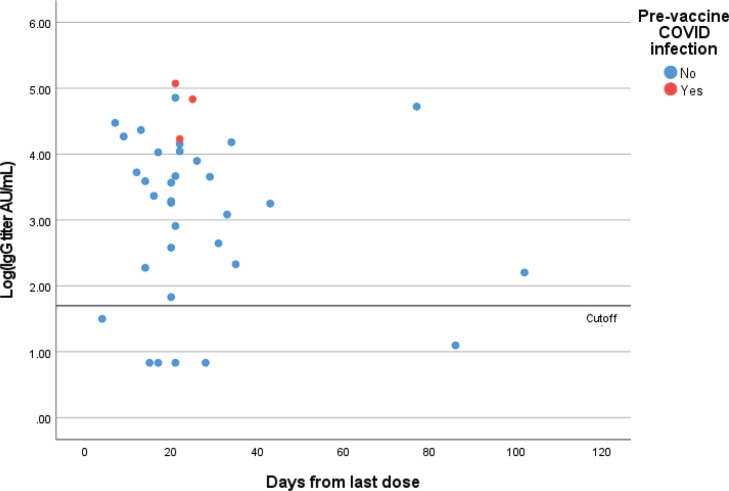
Quantitative IgG titers (logarithmic scale) for patients who received two doses of the vaccine. Each point represents one patient.

**Table 1.** Clinical characteristics of the study cohort.

*N*	50
Age, years, median	64.5
**Gender**	
Male	24 (48%)
Female	26 (52%)
**Type of malignancy**	
Breast	12 (24%)
Lung	8 (16%)
Gastrointestinal	21 (42%)
Genitourinary	3 (6%)
Haematologic	6 (12%)
**Types of treatment**	
Chemotherapy	20 (40%)
Immunotherapy	6 (12%)
Chemotherapy + immunotherapy	5 (10%)
Chemotherapy + anti VEGF	5 (10%)
Anti CD20	2 (4%)
Anti CD38 + proteasome inhibitor	2 (4%)
Anti HER-2	3 (6%)
Chemotherapy + anti HER-2	2 (4%)
Endocrine + CDK4/6 inhibitor	1 (2%)
Anti EGFR	1 (2%)
Chemotherapy + anti EGFR	2 (4%)
Bispecific T cell engager	1 (2%)
**Pre-vaccine COVID infection**	
No	43 (86%)
Yes	7 (14%)
**Vaccine information**	
One dose	14 (28%)
Two doses	36 (72%)
Days between last dose and IgG measurement, median	20

VEGF: vascular endothelial growth factor; HER: human epidermal growth factor receptor; CDK: cyclin-dependent kinase;

EGFR: epidermal growth factor receptor
